# Structural and functional analysis of the active cow rumen’s microbial community provides a catalogue of genes and microbes participating in the deconstruction of cardoon biomass

**DOI:** 10.1186/s13068-024-02495-4

**Published:** 2024-04-08

**Authors:** Andrea Firrincieli, Andrea Minuti, Martina Cappelletti, Marco Ferilli, Paolo Ajmone-Marsan, Paolo Bani, Maurizio Petruccioli, Antoine L. Harfouche

**Affiliations:** 1 Department for Innovation in Biological, Agro-Food and Forest Systems, University of Tuscia, Via San Camillo de Lellis Snc, 01100 Viterbo, Italy; 2 Department of Animal Science, Food and Nutrition, Faculty of Agriculture, Food and Environmental Sciences, Università Cattolica del Sacro Cuore, Via Emilia Parmense 84, 29122 Piacenza, Italy; 3 Department of Pharmacy and Biotechnology, University of Bologna, Via Irnerio 42, 40126 Bologna, Italy; 4 Molecular Genetics and Functional Genomics, Ospedale Pediatrico Bambino Gesù, IRCCS, 00146 Rome, Italy; 5 CREI - Romeo and Enrica Invernizzi Research Center On Sustainable Dairy Production, Università Cattolica del Sacro Cuore, Via Emilia Parmense, 84, 29122 Piacenza, Italy

**Keywords:** Cow rumen microbiome, Metatranscriptomics, *Treponema*, Carbohydrate active enzymes, *Ruminococcus*, *Neocallimastigaceae*, *Cynara cardunculus*

## Abstract

**Background:**

Ruminal microbial communities enriched on lignocellulosic biomass have shown considerable promise for the discovery of microorganisms and enzymes involved in digesting cell wall compounds, a key bottleneck in the development of second-generation biofuels and bioproducts, enabling a circular bioeconomy. Cardoon (*Cynara cardunculus*) is a promising inedible energy crop for current and future cellulosic biorefineries and the emerging bioenergy and bioproducts industries. The rumen microbiome can be considered an anaerobic “bioreactor”, where the resident microbiota carry out the depolymerization and hydrolysis of plant cell wall polysaccharides (PCWPs) through the catalytic action of fibrolytic enzymes. In this context, the rumen microbiota represents a potential source of microbes and fibrolytic enzymes suitable for biofuel production from feedstocks. In this study, metatranscriptomic and 16S rRNA sequencing were used to profile the microbiome and to investigate the genetic features within the microbial community adherent to the fiber fractions of the rumen content and to the residue of cardoon biomass incubated in the rumen of cannulated cows.

**Results:**

The metatranscriptome of the cardoon and rumen fibre-adherent microbial communities were dissected in their functional and taxonomic components. From a functional point of view, transcripts involved in the methanogenesis from CO_2_ and H_2_, and from methanol were over-represented in the cardoon-adherent microbial community and were affiliated with the *Methanobrevibacter* and *Methanosphaera* of the *Euryarchaeota* phylum. Transcripts encoding glycoside hydrolases (GHs), carbohydrate-binding modules (CBMs), carbohydrate esterases (CEs), polysaccharide lyases (PLs), and glycoside transferases (GTs) accounted for 1.5% (6,957) of the total RNA coding transcripts and were taxonomically affiliated to major rumen fibrolytic microbes, such as *Oscillospiraceae*, *Fibrobacteraceae*, *Neocallimastigaceae*, *Prevotellaceae, Lachnospiraceae,* and *Treponemataceae*. The comparison of the expression profile between cardoon and rumen fiber-adherent microbial communities highlighted that specific fibrolytic enzymes were potentially responsible for the breakdown of cardoon PCWPs, which was driven by specific taxa, mainly *Ruminococcus, Treponema,* and *Neocallimastigaceae*.

**Conclusions:**

Analysis of 16S rRNA and metatranscriptomic sequencing data revealed that the cow rumen microbiome harbors a repertoire of new enzymes capable of degrading PCWPs. Our results demonstrate the feasibility of using metatranscriptomics of enriched microbial RNA as a potential approach for accelerating the discovery of novel cellulolytic enzymes that could be harnessed for biotechnology. This research contributes a relevant perspective towards degrading cellulosic biomass and providing an economical route to the production of advanced biofuels and high-value bioproducts.

**Supplementary Information:**

The online version contains supplementary material available at 10.1186/s13068-024-02495-4.

## Background

The development of economical and sustainable biomass conversion technologies for the production of lignocellulosic biofuels is needed to meet the one billion gallons renewable fuel requirement set by the U.S. Environmental Protection Agency in the 2013 Clean Air Act. Lignocellulosic biomass feedstocks, derived from farming dedicated energy crops and agro-based industries as waste by-products, have attracted increasing attention in the production of bio-based materials, chemical products, and biofuels [1].

Lignocellulosic biomass is outside the human food chain and its energetic content exceeds by many times the world’s basic energy requirements [2]. Lignocellulosic biomass is primarily composed of both complex matrices of polysaccharides (cellulose and hemicellulose) and lignin, which are the main constituents of plant cell walls. Because of this, they are together also called plant cell wall polysaccharides (PCWPs). Each of these components has a significant industrial use [3] for instance, lignin is used in the production of sulfur-free solid fuels, sub-bituminous coal, phenolic compounds, and natural binders with potential applications in agricultural practices. The chemical degradation of hemicellulose produces the solvent furfural, which is used at an industrial scale for the production of nylon-6, nylon-6,6, bio-plastics, and resins, while xylose, a sub-product of hemicellulose hydrolysis, can be used for the synthesis of bioethanol, xylitol, and organic acids [3]. In addition, valuable chemicals such as ethanol, lactic acids, acetone, and butanol are obtained as final products of cellulose fermentation [3]. However, due to its physicochemical composition and structural features, lignocellulose is recalcitrant to biological conversion, resulting in a high cost for pretreatment and enzymatic hydrolysis [4–7]. Therefore, several studies have focused on the discovery of efficient and economically viable enzymes and microorganisms that can be exploited at an industrial scale to significantly lower the cost of lignocellulosic biomass conversions [7–9].

Cardoon (*Cynara cardunculus*) is a perennial thistle tribe (*Cynareae*) of the sunflower family (*Asteraceae*) well-adapted to the Mediterranean climate with low water and fertilizers [10]. In addition, *C. cardunculus* can persist for over 10 years, re-sprouting annually from its large perennial taproot [11]. Therefore, cardoon is a suitable bioenergy crop for cultivation on marginal lands. In terms of productivity, cardoon can provide up to 30.0 t/ha year^−1^ (dry matter) of lignocellulosic biomass, which can be used either as a source of bioenergy and/or as raw material for paper pulp production [12–15]. Seeds obtained from the capitula are potentially used for oil and biodiesel production [10], while stalks can be used for paper production [16] or biogas [14]. Cardoon also has high nutritive and energy values, and has been recently proposed as alternative feedstuff ruminants [17, 18]. In addition, cardoon polysaccharides can be degraded to monosaccharides for bioethanol production [19, 20]. Such valuable end-products derive from the deconstruction of PCWPs, and efforts are put into the identification of enzymes and microorganisms that efficiently convert biomass into biofuels and bio-based materials [8, 9, 21]. Understanding the activity of the diverse enzymatic classes that act on the different components of the plant’s cell walls (cellulose, xylans, mannans, pectic substances, and phenylpropanoid polymers) is needed to achieve complete degradation of PCWPs [9]. In addition, as the chemical–physical characteristics of biomass feedstock vary widely, the optimization of the PCWPs degradation may require the combined action of enzymes that synergistically deconstruct each lignocellulosic fraction [22, 23]. Therefore, the development of optimized enzymatic cocktails delivering the best performances in the conversion of lignocellulose into valuable end-products still represents a major challenge in the applicability of such processes at an industrial scale [8]. In the last decades, with the rapid evolution of next-generation sequencing (NGS) technologies, several studies were focused on the taxonomic and functional analysis of microbial communities colonizing biological niches, where the deconstruction of plant cell wall fibers is the central metabolism, e.g., termite gut, soil litter, and animal rumen [8, 24–28]. In this respect, the cow rumen represents a biological system in which microbes are specialized in the degradation of PCWPs. The rumen acts as a highly specialized bioreactor, where PCWPs are efficiently deconstructed via the combined action of enzymes produced by resident microorganisms [25]. Therefore, the study of the rumen microbiota would allow us to define that combination of enzymes and microorganisms that synergistically and optimally deconstructs PCWPs of such promising bio-energy crop, *C. cardunculus.*

The aim of this work was to compare the taxonomic and functional profiles of two microbial communities selected in the rumen environment. The first microbial community was selected by cardoon substrate incubated in the cow rumen within nylon bags, and considered enriched with microbes able to colonize and proliferate on the cardoon biomass up to 48 and 96 h incubation. The second microbial community was obtained from microbes proliferating in association with the solid fraction of the rumen. This microbial community was not selected towards a specific biomass because the forage is composed of different substrates (Additional file 1: Table S1) and subjected to daily turnover through feeding. By comparing these two microbial community, metatranscriptomic analyses of 16S ribosomal RNA (rRNA) and total RNA were performed to get insights into the microbes and enzymes mostly active on cardoon substrate and therefore applicable to PCWPs digestion processes. In particular, the taxonomic affiliation of the genes encoding for glycoside hydrolases (GHs), carbohydrate-binding motif (CBM), polysaccharide lyases (PLs), and carbohydrate esterases (CEs) has been assessed and the predominant genera involved in PCWPs degradation were identified. The expression profile of Carbohydrate-Active enZymes (CAZy) enzymes families was compared between cardoon- and rumen fibre-adherent microbial communities and the deconstruction of cardoon PCWPs was attributed to specific fibrolytic enzymes suitable for saccharification processes, whose expression was ascribed to specific taxonomic groups.

## Results

### In vitro and in vivo digestibility of cardoon biomass

#### In situ digestibility of cardoon biomass using the nylon bag technique

**Fig. 1:**
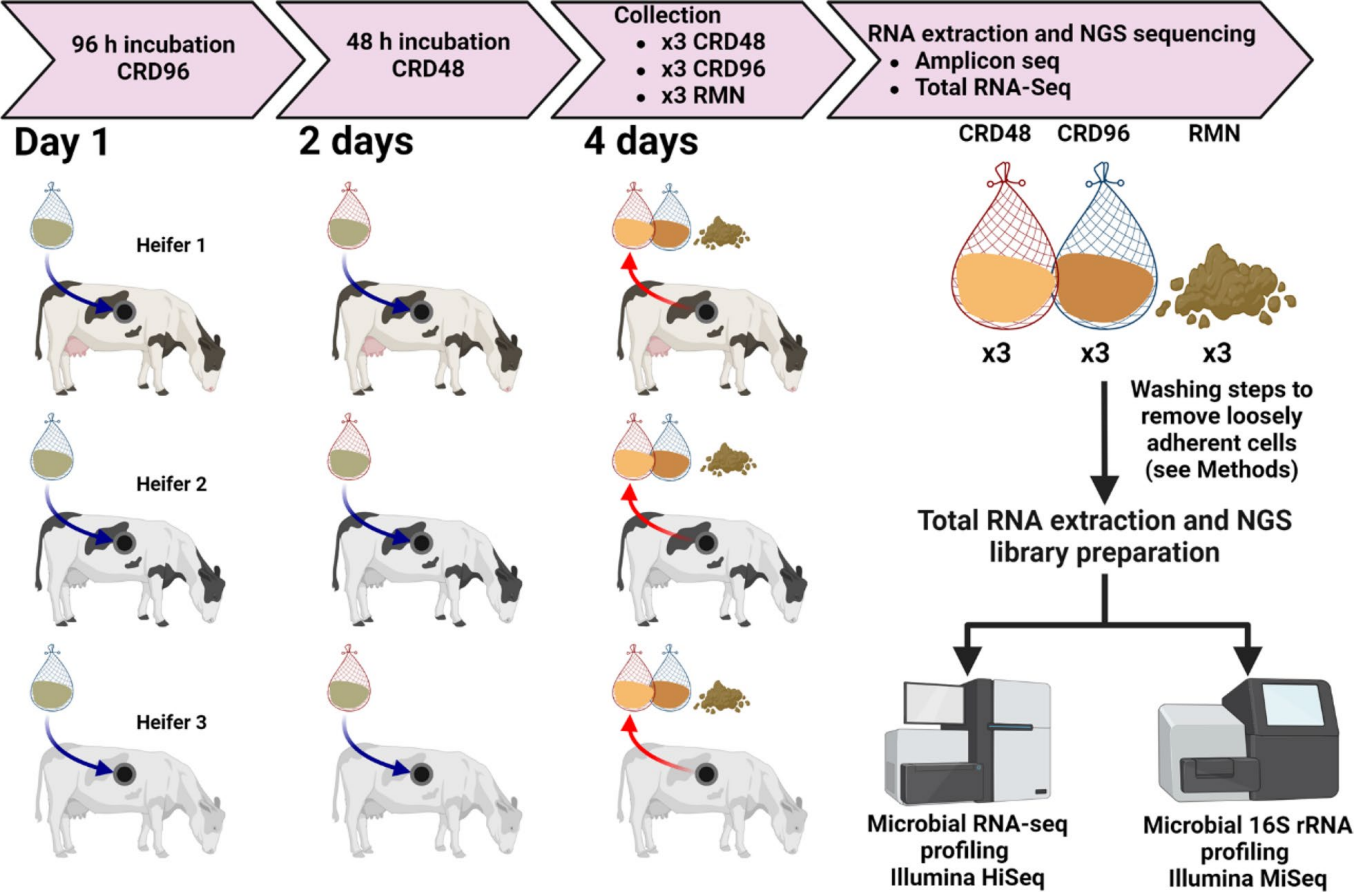
Schematic of the sample preparation, collection, and NGS strategies employed in this study

Six weeks before the in situ rumen incubation of cardoon nylon bags, three rumen-fistulated heifers were fed with a mixed diet containing 6 kg day^−1^ of grass hay, 2 kg day^−1^ of cardoon, and 1 kg day^−1^ of concentrate composed of decorticated sunflowers, soy beans, and wheat bran (Additional file 1: Table S1). In terms of chemical composition, cardoon and grass hay, the latter representing the main fibrous fraction of the animal diet, were similar except in the crude protein fraction which was almost double in grass hay (84.80 g kg^−1^ of dry matter) compared to cardoon (44.72 g kg^−1^ of dry matter). The introduction of small amount of cardoon into the animal diet was performed in order to stimulate the rumen microbiota this biomass, and ensuring that the nylon bags system will enrich for microbes actually able to colonize the cardoon and deconstruct its PCWPs fraction. After 6 weeks, the nylon bags containing only air-dried cardoon were incubated into the rumen of the three rumen-fistulated heifers to enrich for lignocellulolytic microorganisms involved in cardoon digestion. The incubation of the nylon bags in the rumen was carried out for a period of time of 48 and 96 h, and the metatranscriptomic profile of the cardoon-enriched microbial community at 48 (CRD48) and 96 h (CRD96) was compared against the metatranscriptome of the microbial community adherent to the solid fraction of the rumen (RMN), the latter collected from the same animal (Fig. 1).

The digestibility and chemical composition of cardoon residue after 48 and 96 h incubation were measured to confirm that the metatranscriptomes of the microbial community enriched in the nylon bags is actively digesting the cardoon biomass. As a measure of the cardoon in situ digestibility, 54.8% and 70.5% of the cardoon dry matter and the 47.4% and 64.5% of initial fibrous fraction (NDF) were digested in the nylon bags after 48 and 96 h of incubation, respectively (Table 1).

**Table 1:** In situ digestibility (g kg^−1^ of initial weight), and chemical composition (g kg^−1^, dry matters basis) of the residue and ruminal solids

Digestibility^1^	Cardoon	
	48 h	96 h
Dry matter	547.82 ± 54.28	704.65 ± 66.36
NDF	473.91 ± 44.64	645.42 ± 53.59

Residue^2^ (chemical composition)	Cardoon			Rumen solid^2^
	Undigested	48 h	96 h	–
NDF	570.73 ± 0.7	697.66 ± 7.98	685.11 ± 5.59	767.44 ± 8.98
Acid detergent fiber (ADF)	458.11 ± 0.08	553.55 ± 4.57	543.84 ± 3.41	534.33 ± 1.32
Acid detergent lignin (ADL)	72.11 ± 2.15	165.89 ± 3.96	178.69 ± 4.52	126.19 ± 2.86
% Lignification (ADL/NDF)	12	24	26	16

^*1*^Cardoon digestibility measured from biomass samples collected from nylon bags at 48 and 96 h incubation are reported in Additional file 1: Table S2

^2^The chemical composition of the rumen solid was measure at the end of the in situ experiment (see Fig. 1 for reference)

With respect to the chemical composition of the cardoon residue in the nylon bags, high concentrations of ADF, ADL, and NDF were measured after 48 h incubation, with 24% increase in the lignification of the NDF fraction of the residue (Table 1). Conversely, no substantial increments in ADL, ADF and NDF were observed between 48 and 96 h incubation (Table 1). The lack of differences in the chemical composition between 48 and 96 h indicates that the microbial community enriched in the nylon bags is associated with the most recalcitrant fraction of the residue, since the less coriaceous fibrous fraction has been already degraded within the 48 h incubation period, but that this population is still active at 96 h of incubation in the rumen, as the digestibility of the cardoon DM increased between 48 and 96 h.

### Taxonomy profiling of the active rumen and cardoon-adherent microbial communities

#### Microbial taxonomy profiling based on 16S rRNA (cDNA) amplicon sequencing data

**Fig. 2:**
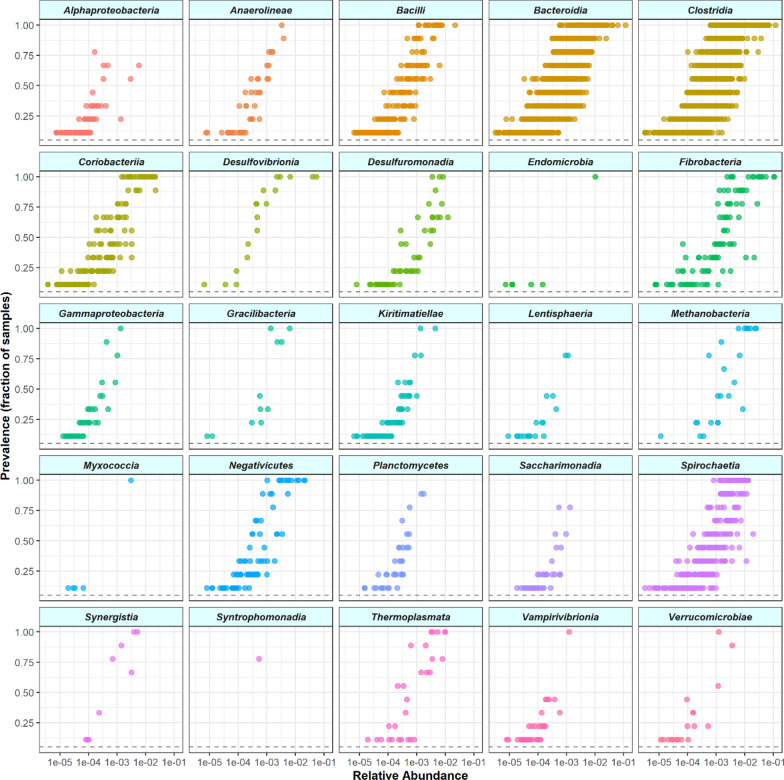
Relative abundance of the top 25 Classes with the highest prevalence across all samples. Only Classes represented by at least one ASV (colored dot) with a prevalence (sample fraction) > 0.75 are shown. The horizontal dashed line indicates a prevalence of 0.1

From the viable microbial community, a total of 6,078 Amplicon Sequence Variants (ASVs) were assembled. *Clostridia*, *Bacteroidia*, *Fibrobacteria*, *Spirocaethia*, *Negativicutes*, and *Coriobacteria* were identified as the major colonizers of the RMN and CRD fibre-adherent microbial communities because represented by a large fraction of ASV with sample prevalence above 0.75 (Fig. 2) (Additional file 1: Table S3).

**Fig. 3:**
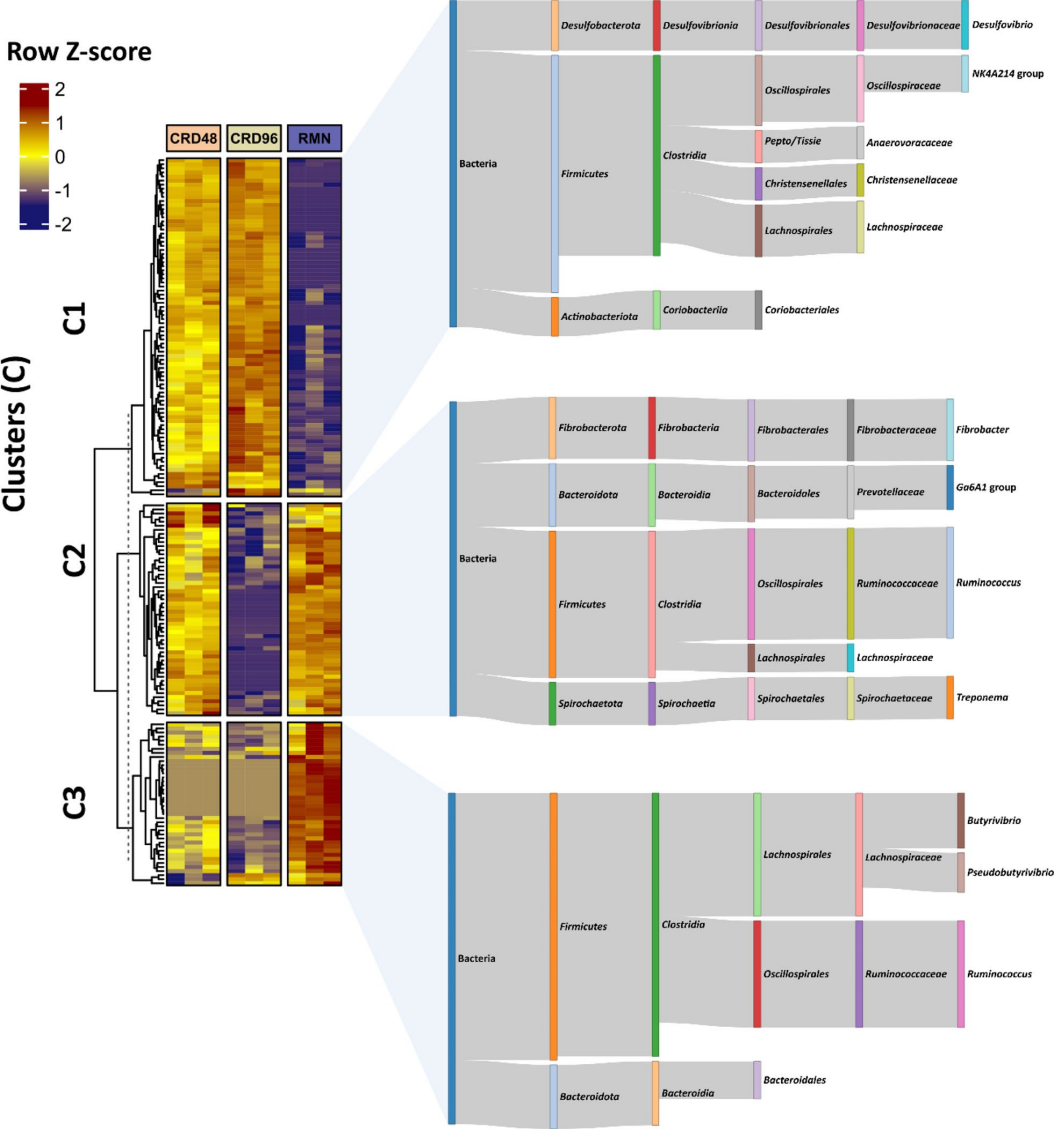
Heatmap clustering and taxonomic profiling of ASV differentially abundant between CRD48, CRD96, and RMN samples. The heatmap scale displays the row Z-score of the log10-transformed abundance data. K-means clustering was performed by setting ‘km = 3’ and the dashed line indicates the separation between the parent dendrogram and the child dendrograms. The sankey diagrams are used show the taxonomic composition of the differentially abundant ASVs. Only taxon with a relative abundance > 1% within each cluster (C1, C2, C3) are shown in the sankey diagram. *Peptostreptococcales–Tissierellales* are indicated as *Pepto/Tissie*

The alpha diversity indices Shannon and Chao1 indicate that the CRD-adherent microbial communities have higher diversity and richness compared to the RMN-adherent microbial communities (Additional file 1: Table S4). Differential abundance analysis identified 175 ASVs showing significant changes between CRD and RMN samples (Additional file 1: Table S5) and organized in three clusters, i.e., C1, C2, and C3 showing distinct abundance profiles across the RMN and CRD-adherent microbial community (Fig. 3).

Particularly, *Butyrivibrio, Pseudobutyrivibrio,* and *Ruminococcus* ASVs were more abundant in the RMN-adherent microbial communities, while a different population of *Ruminococcus*, along with *Fibrobacter*, *Prevotellaceae, Lachnospiraceae,* and *Treponema* ASVs, were more abundant in the RMN-adherent microbial communities and CRD48 samples (Fig. 3). Finally, ASVs affiliated to the N4KA214 group (*Oscillospiraceae*), *Desulfovibrio, Lachnospiraceae, Christensenellaceae, Anaerovoraceae,* and Coriobacteriales were significantly more abundant in the CRD-adherent microbial communities samples (CRD48 and CRD96) (Fig. 3).

### Microbial taxonomy profiling based on RNA-Seq data

**Fig. 4:**
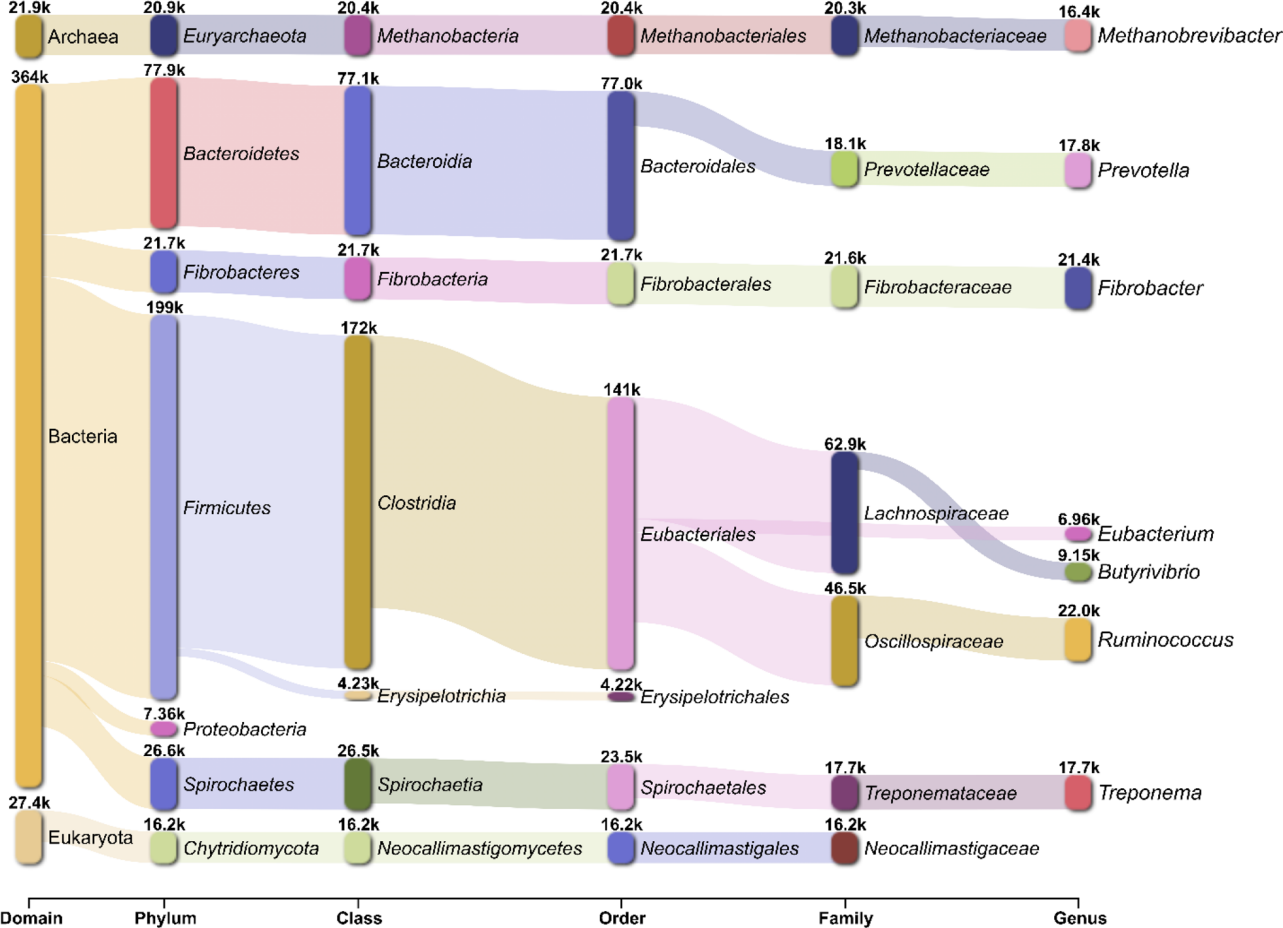
Sankey diagram representing the taxonomic classification of transcripts of the CRD- and RMN-adherent microbial communities. Within each taxonomical rank, only the top 7 most abundant taxa are shown. On each node, the number of transcripts classified within a given taxon is displayed

RNA-seq reads from CRD and RMN samples were assembled into a reference metatranscriptome of 465,272 transcripts with an average length of 995.6 bp and an N50 1029 bp. A total of 363,975 transcripts were assigned to Bacteria (78.2%), while 21,895 and 27,679 transcripts were taxonomically affiliated to Archaea (4.70%) and Eukaryota (5.94%), respectively (Fig. 4**;** Additional file 1: Table S6).

**Fig. 5:**
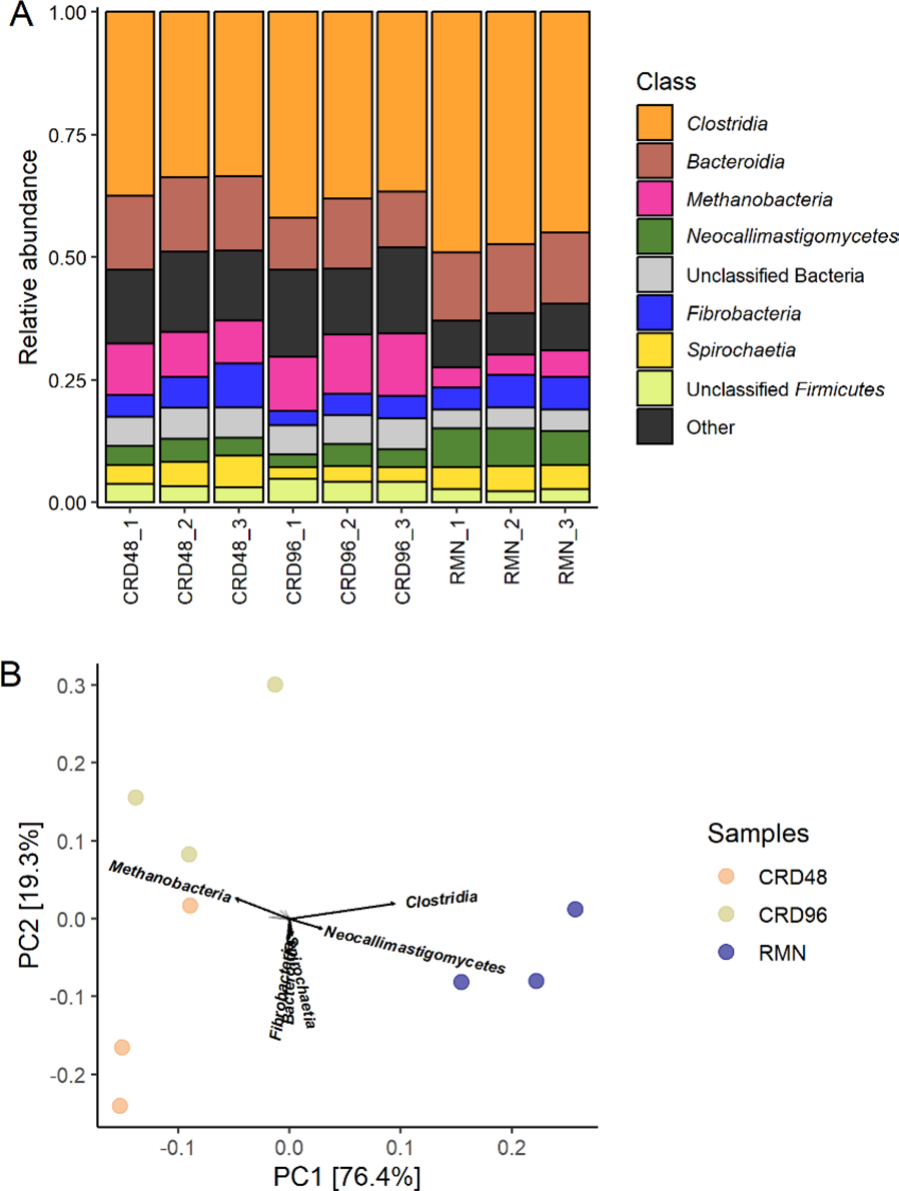
Class level relative abundance and PCA analysis of the CRD and RMN-adherent microbial communities. **A** Relative abundance data are shown only for the top 8 most abundant Classes. **B** principal component analysis based on relative abundance data; arrows represent the top 6 Classes that mostly contribute to the separation between samples

In agreement with 16S rRNA data, a large proportion of Bacteria transcripts were affiliated with *Firmicutes* (42.7%), followed by *Bacteroidetes* (16.7%), *Spirochaetes* (5.71%), and *Fibrobacteres* (4.66%) (Fig. 4**;** Additional file 1: Table S6). In addition, the RNA-seq analysis showed that Archaea and Eukaryota transcripts were affiliated with *Methanobacteriaceae* (4.36%) and *Neocallimasticaceae,* respectively (3.47%) (Fig. 4**;** Additional file 1: Table S6). Samplewise relative abundance analysis of the metatranscriptome coding fraction (mRNA) identifies *Clostridia*, *Bacteroidia*, *Methanobacteria*, *Neocallimasticomycetes*, *Spirochaetia*, and *Fibrobacteria* as the most abundant and active taxa in the CRD- and RMN fibre-adherent microbial communities (Fig. 5A).

However, PERMANOVA analysis on Bray − Curtis dissimilarities highlights significant changes in terms of composition between the CRD- and RMN-adherent microbial communities (R^2^ = 0.659; *P* = 0.014) with *Methanobacteria*, *Clostridia*, and *Neocallimasticomycetes* mostly contributing to the differentiation between the two communities in the ordination plot (Fig. 5B).

### Functional profiling of the cardoon- and rumen fiber-adherent microbial communities

The functional profiling of the transcripts representing the cardoon- and rumen fibre-adherent microbial community was performed to gain further insights into the most active taxa contributing to the degradation of cardoon PCWPs, and the methanogens boosting the cellulolytic activity of the fibre-degrading microbial community [29, 30].

**Fig. 6:**
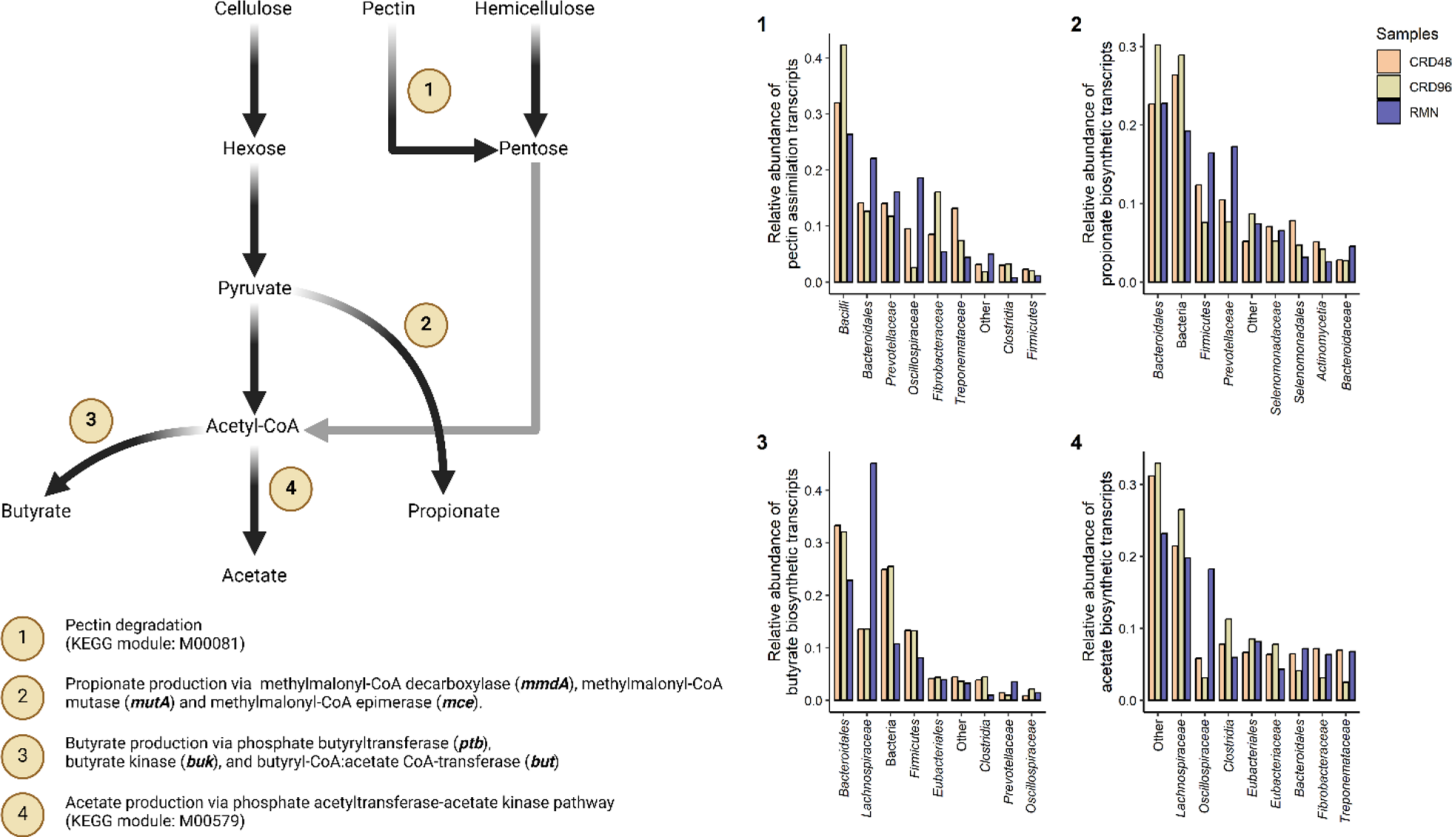
Fig. 6 Transcriptionally active taxon in the expression of transcripts involved in pectin assimilation and VFAs biosynthesis. Bar plots show the relative abundance calculated from the total count of reads mapping to the metatranscriptome-assembled transcripts involved in pectin degradation (1), propionate biosynthesis (2), butyrate biosynthesis (3), and acetate biosynthesis (4). Relative abundance data are shown only for the top 8 most abundant taxon down to the family level

A total of 236,836 transcripts were assigned to 97,946 phylogenetically refined orthologous groups (SdO, seed orthologs) (Additional file 1: Table S7). Functional annotation of SdO-associated transcripts identified 5,693 unique KEGG Orthologs (KOs) terms, some of them being associated with metabolic modules involved in pectin assimilation, and fermentation of sugars (hexoses and pentoses) into the volatile fatty acids (VFA) butyrate, propionate, and acetate (Fig. 6).

Despite a large fraction of transcripts remaining classified at high taxonomic levels (i.e., Domain, Phylum, Class, and Order), major fibre-degrading taxa, such as *Oscillospiraceae, Lachnospiraceae, Treponemataceae,* and *Prevotellaceae,* resulted transcriptionally active in consideration of genes involved in the biosynthetic pathway of VFAs, and pectin assimilation (Fig. 6).

Because less diverse than its bacterial counterpart, the transcripts involved in methane metabolism were only affiliated with the methanogens *Methanobrevibacter* and *Methanosphaera.* In particular, transcripts associated with methane production from CO_2_ and H_2_ were affiliated with *Methanobrevibacter*, while the utilization of the methanogenic substrate methanol could be attributed to members of the *Methanosphaera* genus (Additional file 2).

**Fig. 7:**
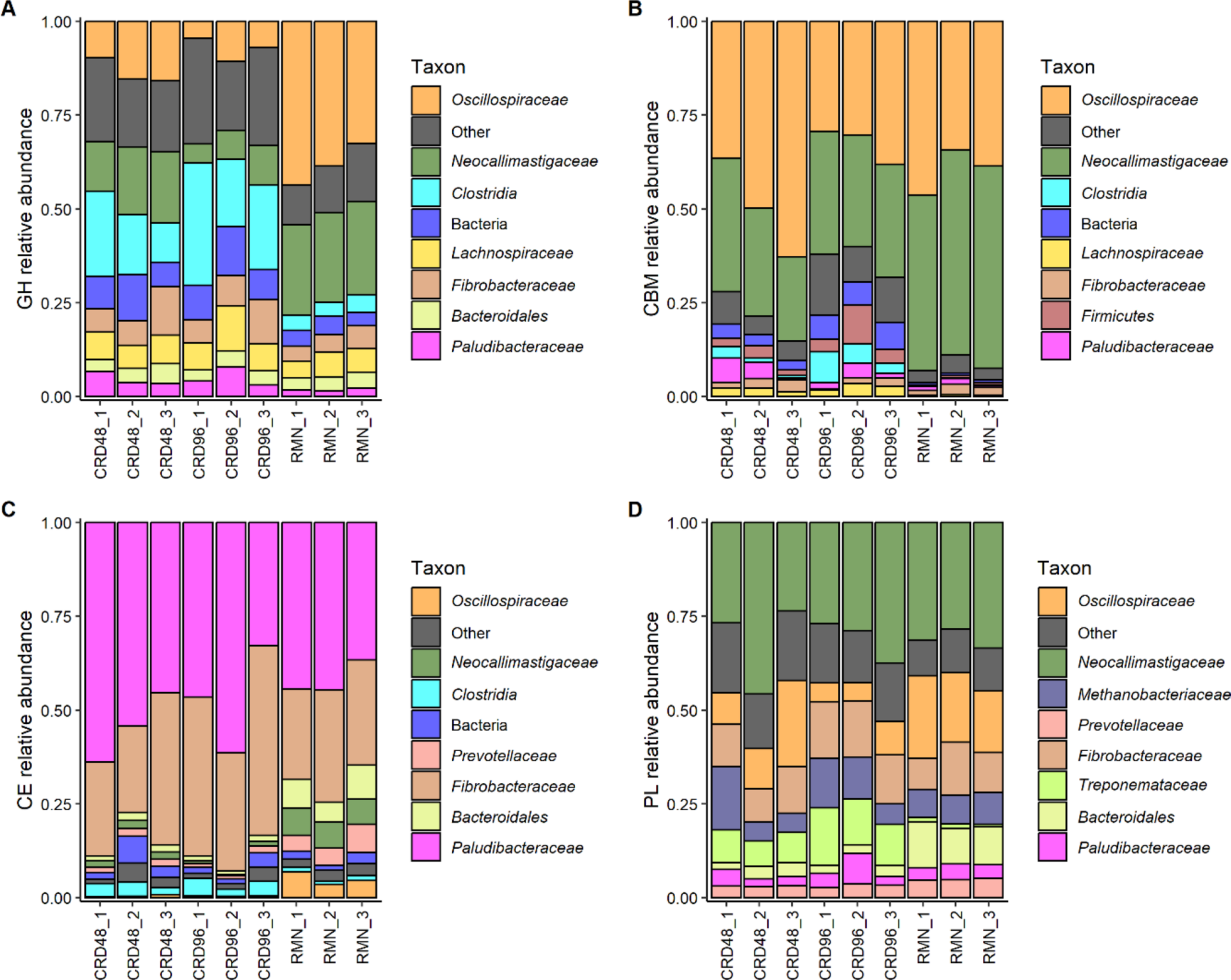
Taxon contribution to the relative expression of the CAZy categories GH, CBM, CE, and PL. **A**–**D** Relative abundance calculated from the total count of reads mapping to the metatranscriptome-assembled transcripts annotated as glycoside hydrolases (GH, **A**), carbohydrate binding modules (CBM, **B**), carbohydrate esterases (CE, **C**) and polysaccharide lyases (PL, **D**). Only the top 8 most abundant taxon contributing to the expression of each CAZy category are shown

The taxonomic affiliation of Carbohydrate active enzymes (CAZy)-coding transcripts was also investigated to identify the taxa and the enzymatic classes directly involved in the deconstruction of cardoon PCWPs. In total, 7,393 transcripts were found to encode for CAZy (Additional file 1: Table S8) of which 790, 234, 4,971, and 962 were annotated with at least one CBM, GH, CE, and PL functional domain, respectively. The highest number of transcripts in CRD and RMN samples with a CBM, GH, and PL domain belonged to *Oscillospiraceae* and *Neocallimasticaceae*, while *Paludibacteraceae* and *Fibrobacteraceae* accounted for the majority of CE transcripts (Fig. 7).

The GH families predominantly present in RMN and CRD samples were GH43 and GH13, accounting, respectively, for 34% and 23% of the total GH transcripts (Additional file 1: Table S9). The domains CBM18, CBM37, and CBM1 were identified in 42%, 15%, and 10% of the transcripts with a CBM domain, while approximately 87% of the CE transcripts were annotated as CE6 (59%), CE11 (14%), and CE12 (13%) (Additional file 1: Table S9). Finally, the most abundant PL families identified in CRD and RMN samples were PL11 (37%), PL1 (34%), and PL9 (14%) (Additional file 1: Table S9). The above fibrolytic enzymes, therefore, represent the enzymatic repertory exploited by the fibre-degrading microbial community associated with both rumen and cardoon biomass.

#### Substrate-specific expression profile of the biomass-adherent microbial community

**Fig. 8:**
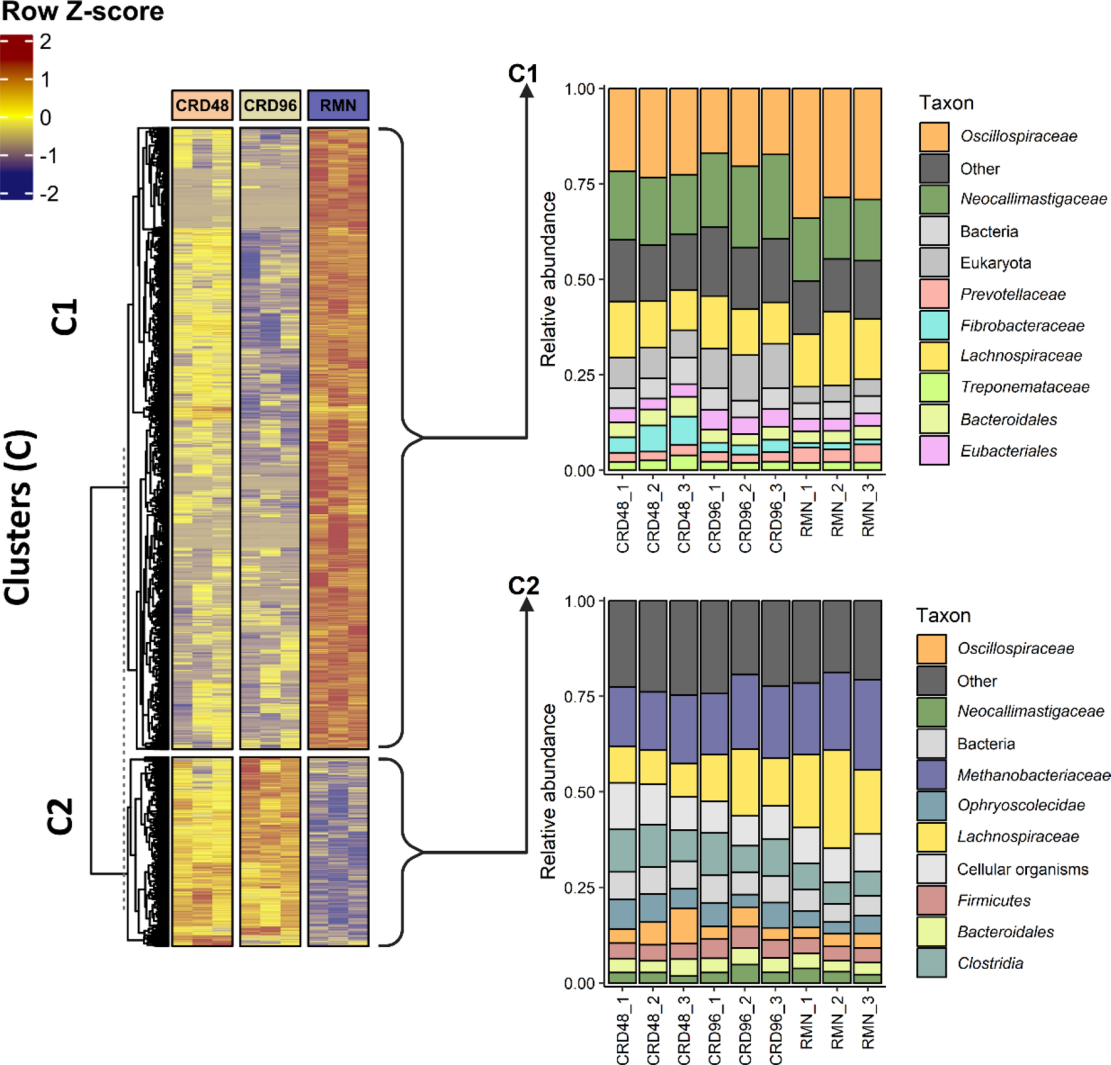
Taxonomic profiling of the differentially expressed seed orthologs between the CRD and RMN-adherent microbial communities. The heatmap scale displays the row Z-score of the CPM log2 transformed counts. K-means clustering on rows was performed by setting ‘km = 2’ and the dashed line indicates the separation between the parent dendrogram and the child dendrogram. Relative abundances were calculated from the total count of reads mapping to the metatranscriptome-assembled transcripts belonging to the differentially expressed seed orthologs in C1 and C2 clusters

A total of 9,647 SdO representative of 44,622 transcripts were differentially expressed (DE) between CRD and RMN (FDR < 0.01) (Additional file 1: Table S10). Overall, the DE SdO clustered in two groups consisting of 7,038 SdO more expressed in RMN samples, and 2,609 SdO more expressed in the CRD fibre-adherent microbial community (Fig. 8).

DE SdO were mostly represented by transcripts affiliated to major fibre-degrading families, i.e., *Oscillospiraceae*, *Lachnospiraceae*, *Neocallimastigaceae*, *Prevotellaceae*, and *Treponemataceae,* while transcripts affiliated to *Methanobacteriaceae* were only detected in the SdO significantly more expressed in the CRD samples (Fig. 8). Functional over-representation analysis indicates that the KEGG modules of the chemotaxis (M00506, CheA–CheYBV two-component regulatory system), multiple sugar transport systems (M00216), eukaryotic ribosome (M00177), F-type ATPase complex (M00157), and prokaryotic succinate dehydrogenase (M00149) were over-represented in the SdO significantly more expressed in the RMN fibre-adherent microbial community (Additional file 1: Table S11). Conversely, the methanogenesis modules (M00356, M00563, M00567, M00357) were over-represented in the SdO more expressed in the CRD fibre-adherent microbial community (Additional file 1: Table S11).

#### Within-taxon differential expression analysis in PCWPs degrading microbes

To account for biases introduced by organism-independent (global) scaling of metatranscriptomic counts data [31, 32], a within-taxon differential expression analysis was performed to better capture the contribution of PCWPs degrading microbes to the deconstruction of cardoon PCWPs. This analysis was conducted to better characterize the repertory of the CAZy-coding transcripts differentially expressed within the major PCWPs degrading families identified in our study, i.e., *Oscillospiraceae*, *Fibrobacteraceae*, *Neocallimastigaceae*, *Prevotellaceae, Lachnospiraceae, Treponemataceae*, and *Paludibacteraceae* (Fig. 4).

*Oscillospiraceae* and the anaerobic fungi *Neocallimastigaceae* were the families showing the highest percentage of transcripts and CAZy-coding transcripts differentially expressed (FDR < 0.05) between RMN and CRD samples, followed by *Treponemataceae*, *Prevotellaceae*, and *Lachnospiraceae* (Table 2).

**Table 2:** Differential expression (DE) analysis of transcripts affiliated with major fibre-degrading families in rumen and cardoon samples

Family	Total mRNA transcripts	Total CAZy transcripts	% of Differentially expressed (DE) transcripts^1^	DE CAZy transcripts^2^
*Neocallimastigaceae*	15,389	644	23% (3572)	27% (178)
*Oscillospiraceae*	46,338	1006	7.5% (3493)	11.5% (116)
*Fibrobacteraceae*	21,557	403	n.d	n.d
*Prevotellaceae*	17,991	252	6.8% (1225)	8.7% (22)
*Lachnospiraceae*	62,729	509	7.2% (4866)	6.6% (34)
*Treponemataceae*	17,666	136	5.9% (1059)	11% (15)
*Paludibacteraceae*	6462	79	n.d	n.d

^1^Complete list of DE transcripts is provided as Additional file 1: Table S12

^2^Complete list of DE CAZy transcripts is provided as Additional file 1: Table S13

n.d. not detected

**Fig. 9:**
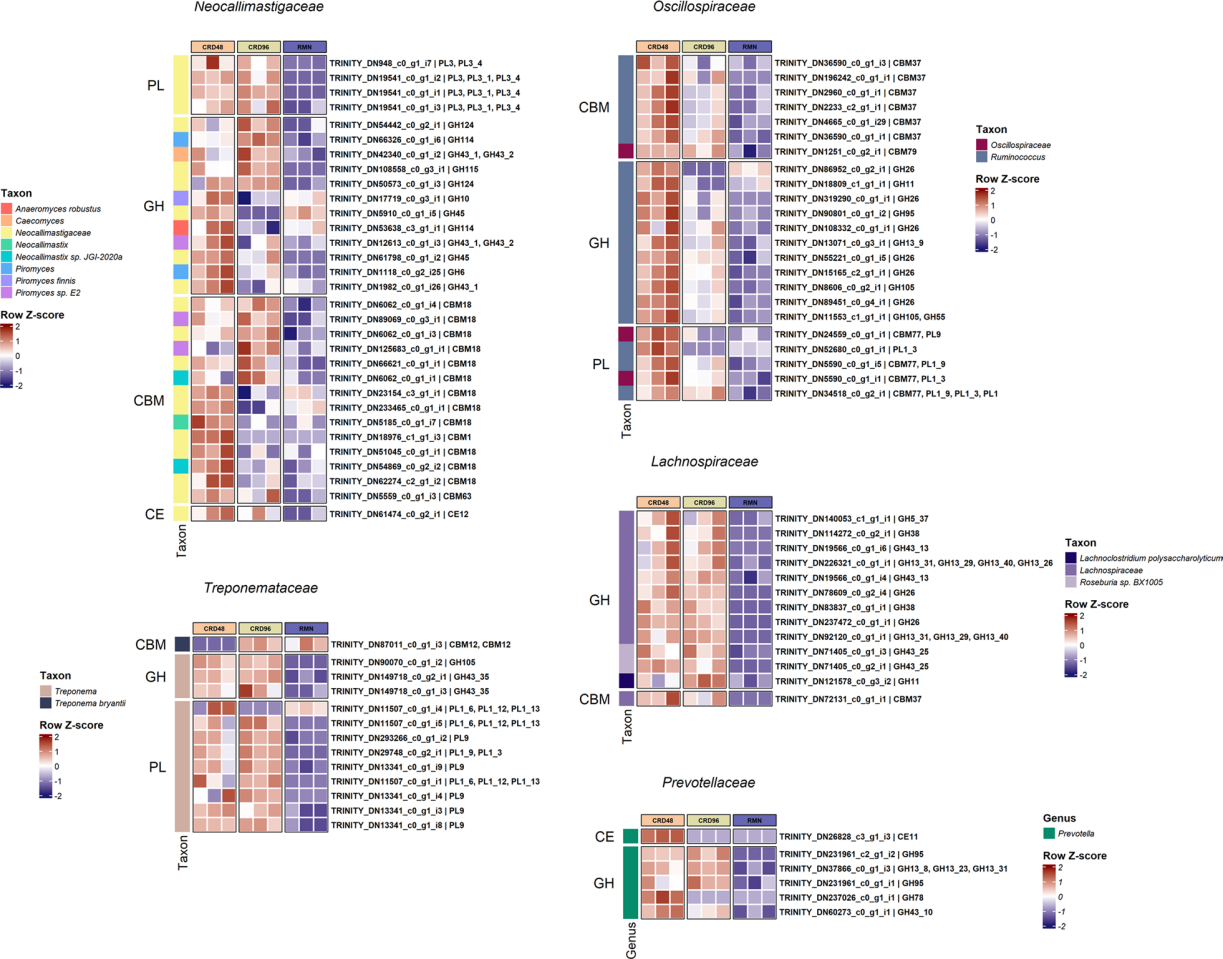
Within-taxon differentially expressed transcripts coding for GH, CE, PL, and CBM fibrolytic enzymes. Only transcripts up-regulated in CRD48 and CRD96 samples with respect to the RMN samples are shown. The heatmap scale displays the row Z-score of the CPM log2 transformed counts

Specific families of carbohydrate-active enzymes were found to be over-represented (adjusted *p* value < 0.1) among the CAZy-coding transcripts significantly more expressed in the CRD-enriched microbial community (Fig. 9). Notably, within this community, *Ruminococcus* (*Oscillospiraceae*) exhibited a preference for overexpressing a set of carbohydrate-active enzymes represented by the GH26, GH105, CBM37, CBM77, and PL1 families, while *Treponema* (*Treponemataceae*) and *Lachnospiraceae* preferentially overexpressed the PL9 and GH26 CAZy-coding transcripts, respectively (Additional file 1: Table S14). These findings suggest that within the CRD-enriched microbial community, *Ruminococcus*, *Treponema*, and *Lachnospiraceae* selectively overexpress a distinct set of carbohydrate-active enzymes involved in the deconstruction of cardoon PCWPs.

Outside the ORA, all major fibre-degrading families, excluding the *Ruminococcus*, overexpressed one or more copies of GH43-coding transcripts in association with the cardoon-adherent microbial community (Fig. 9). In *Ruminococcus,* on the other hand, a large fraction of the GH-coding transcripts over-expressed in the cardoon-adherent microbial community were annotated as GH26, a family that includes members catalytically active towards mannans and xylans (Fig. 9).

Some degree of conservation regarding the over-expression of CAZy-coding transcript within the cardoon-adherent microbial community was also observed for the PL and CBM families which resulted also affiliated to *Ruminococcus*. For instance, *Oscillospiraceae/Ruminococcus* CBM- and PL-coding transcripts more expressed in the cardoon-adherent microbial samples were exclusively annotated as CBM77 and CBM37, and PL1 and PL9, respectively (Fig. 9). Similarly, *Treponema* PL-coding transcripts with a significantly higher expression in both CRD48 and CRD96 samples were also exclusively annotated as PL1 and PL9. Less diversified was also the pool of *Neocallimastigaceae* CBM- and PL-coding transcripts overexpressed in CRD fibre-adherent microbial community. Most of these transcripts were not classified down to genus/species level and belonged almost exclusively to the CBM18 family, which comprises only members with a chitin-binding activity, and the pectate lyases of the PL3 family. Finally, unlike for *Ruminococcus*, the repertory of *Neocallimastigaceae* GH-coding transcripts was much more diversified, including enzymes with different substrate specificity towards xylans (GH10, GH43), polygalactosamine (GH114), 4-O-methyl D-glucuronic acid (GH115), and glucans (GH124, GH45, GH6) (Fig. 9).

What is really interesting from the taxon-specific expression profiles shown in Fig. 9 is that the expression profile of *Ruminococcus* and, to less extent, *Neocallimastigaceae* CAZy-coding transcripts drop after 48 h, while for some taxa, i.e., *Lachnospiraceae* and *Treponema*, the expression of the CAZy-coding transcripts persist at higher level up to 96 h incubation in association with the most recalcitrant fraction of the cardoon residue.

## Discussion

Cardoon is a promising bioenergy crop with multiple end-use applications in bioenergy and bioproduct industries, and as an alternative feedstuff for ruminants [18, 33].

In the present work, 16S rRNA cDNA amplicon sequencing and RNA-Seq metatranscriptomic were performed to detect, within the rumen microbiome, the most active microbial community and enzymes enriched in the nylon bags and involved in the deconstruction of cardoon PCWPs. The in situ experiments were conducted on three cannulated heifers fed on a daily (d) basis with a diet containing grass hay (6 kg d^−1^), cardoon (2 kg d^−1^), and concentrate (1 kg d^−1^). Because dietary factors, such as the chemical composition of the forage, can affect the ruminal microbiota composition, these can also potentially influence the rate of digestion of the biomass tested in the nylon bags [34]. Therefore, to stimulate the rumen microbiota to degrade the biomass tested in the nylon bags, a small fraction of cardoon was introduced into the animals diet. This can be considered a standard good practice for the in situ technique [35]. Indeed, without feeding the animal with a small amount of cardoon, the rumen microbiota would only partially respond to the tested biomass with the consequence of “enriching” the nylon bags with microbes that poorly degrade the cardoon. In addition, we were also interested in the enrichment of a microbiota that, with its enzymatic repertory, could act on a cardoon biomass incubated long enough in the rumen that could represent a matrix of difficult degradation. For this purpose, if the nylon bags are incubated into the rumen up to 96 h, this would allow the enrichment of a microbiota best suited to grow on a substrate which can be considered “exhausted” by the common microorganisms that preferentially proliferates associated to solid fraction of the rumen which instead is subjected to daily turnover through feeding. By measuring the chemical composition of the residue in the nylon bags we confirmed that the biomass incubated in the rumen was already exhausted, since no substantial increment in the NDF, ADF, and ADL fractions was observed between 48 and 96 h (Table 1).

### The metabolic potential of the cardoon enriched microbial community

First, taxonomic analysis using both 16S rRNA cDNA amplicon sequencing and RNA-Seq-based metatranscriptomic identified *Clostridia*, *Fibrobacteria*, *Spirocaethia*, and *Bacteroidia* as major the fibre-degrading Classes [36–39], and *Methanobacteria* as the most active methanogenic Archaea (Figs. 2, 3, 4, 5A). Further insights on the eukaryotic community colonizing CRD and RMN samples were obtained from RNA-Seq-based metatranscriptomic analysis, identifying, and expected, *Neocallimastigomycetes* as the eukaryotes colonizing both substrates (Fig. 4). Overall, the most striking differences observed between RMN and CRD samples were the overexpression of transcripts involved in methylotrophic (KEGG modules M00356 and M00563) and hydrogenotrophic (KEGG modules M00567) methanogenesis pathways in the CRD-adherent microbial community (Fig. 8; Additional file 1: Table S10–11). Such increase in the transcriptional activity of methanogens indicates an active production of methanogenic substrates such as CO_2_ and H_2_, methanol, and VFAs carried out by fibre-degrading taxa growing in association with the cardoon residue in the nylon bags. The main methanogens identified in our study were affiliated to *Methanobrevibacter* and *Methanosphaera*, which can perform methanogenesis from the substrates CO_2_ and H_2_, and methanol, respectively (Additional file 2) [40, 41]. In the rumen, one of the primary sources of methanol is pectin fermentation, as a result of a demethoxylation reaction catalyzed by pectin methyl esterases (PME, K01051) [42]. In agreement with a previous study [42], we found that in CRD and RMN samples, the major pectin degraders were represented by *Bacteroidales* (*Bacteroides*), *Prevotellaceae*, *Oscillospiraceae* (*Ruminococcus*), and *Fibrobacteraceae* (Fig. 6). Similar to methanol, bacterial fermentation of polysaccharides into the VFAs butyrate and acetate also promotes the *Methanobrevibater* methanogenesis [43], via acetate assimilation through an incomplete reductive tricarboxylic acid cycle [41]. In this respect, *Lachnospiraceae* were the only taxonomic group showing a higher relative abundance of the transcripts involved in acetate production in cardoon biomass (Fig. 6), which is in agreement with previous studies recognizing *Lachnospiraceae* as one of the major acetogens in the bovine rumen [44, 45].

### GH families catalyzing the cellulose and hemicellulose breakdown in cardoon biomass

The fibrolytic enzymes coding transcripts detected in our samples were mostly restricted to a limited number of taxonomic groups represented by known PCWPs degrading-families, i.e., *Oscillospiraceae*, *Fibrobacteraceae*, *Prevotellaceae*, *Lachnospiraceae*, *Paludibacteraceae*, *Treponemataceae* and *Neocallimastigaceae* (Fig. 7). While the data reported in this work highlighted that the majority of the GH families transcripts were annotated as GH43 and GH13 (Additional file 1: Table S9), *Ruminococcus,* and to less extent *Treponema*, preferentially overexpressed GH26-coding transcripts only within the CRD-enriched microbial community (Fig. 9). The GH43 family currently includes α-l-arabinofuranosidase, β-d-xylosidase, α-l-arabinanase, and β-d-galactosidase enzymes which catalyze the debranching and degradation of hemicellulose and pectin [46]. Members of the GH43 family also possess multifunctional exo-β-xylosidase, endo-xylanase, and α-arabinofuranosidase catalytic activities [47, 48]. In our study, *Prevotella* and *Neocallimastigaceae* were the only PCWPs degrading families showing GH43 transcripts significantly over-expressed in CRD samples (Fig. 9)*.* In particular, the *Neocallimastigaceae* GH43 were classified as members of the GH43 subfamilies 1 (GH43_1) and 2 (GH43_2), which are catalytically active towards birchwood xylan, beta-xylan, linear arabinan, and pNP-α-L-arabinofuranoside [46, 49]. Compared to the subfamilies GH43_1 and GH43_2, the GH43_10 subfamily over-expressed by *Prevotella* (Fig. 9) possesses instead a much broader substrate specificity, being catalytically active against wheat and alpha–alpha arabinoxylans, sugar beet arabinan, and methylumbelliferyl-xylose [46]. The GH13 was the second most abundant GH family detected in our metatranscriptome (Additional file 1: Table S9), and it is considered one of the largest families of glycoside hydrolases [50]. The GH13 overexpressed in CRD samples resulted taxonomically affiliated to *Lachnospiraceae* and *Prevotella,* and significantly aligned against subfamilies (GH13_8, GH13_31, GH13_29, and GH13_40) of glucan branching enzymes, amylases, and glucan 1,6-α-glucosidases [50]. Finally, the majority of glycoside hydrolases overexpressed CRD samples by *Ruminococcus* belonged to the GH26 family. Members of this family are classified as hemicellulases represented by endo-β-1,4-mannanases and are typically produced by members of the genera *Ruminococcus*, *Prevotella*, and *Fibrobacter* [25]. However, a substantial drop in the GH26 expression was observed from 48 to 96 h incubation (Fig. 9), suggesting that *Ruminococcus* was transcriptionally less active in association with the most recalcitrant fraction of the cardoon residue.

### Pectin breakdown in cardoon biomass

Pectin is a component of the middle lamella and primary cell wall of higher plants and is mainly composed of galacturonate units. Pectin degradation is carried out by the combined action of two enzymatic classes: methylesterases, which remove the methoxyl groups from pectin with the consequent depolymerization of the demethylated poly-galacturonate, and pectin lyases, which depolymerize pectin. Members of *Bacteroidetes* and *Spirochetes* (*Treponema*) were previously identified as the major pectinolytic organisms in host microbiomes [51–54]. In our study, transcripts annotated as PLs were affiliated with *Neocallimastigaceae*, *Oscillospiraceae, Fibrobacteraceae*, and *Treponemataceae* (Fig. 7). The majority of the PL transcripts overexpressed in CRD samples belonged to the polygalacturonate lyase families PL1, PL3, and PL9 [55, 56]. The PL3 family transcripts were affiliated with *Neocallimastigaceae,* whereas PL1 and PL9 belonged to *Ruminococcus*/*Oscillospiraceae* and *Treponema* (*Treponemataceae*). To date, while no functional studies have been carried out for PL1 and PL9 in *Treponemataceae*, members of this taxon were found to be particularly responsive to a pectin-rich diet and supposedly play an important role in pectin breakdown in the rumen [57, 58]. On the other hand, the *Oscillospiraceae* PL1 and PL9 were functionally characterized in *Ruminococcus* for their capacity to degrade pectin in a wide range of substrates. In particular, *Ruminococcus* PL1 and PL9 found in association with a CBM77 domain (CBM77_PL1/9_) are catalytically active towards homopolygalacturonan with low degrees of methyl esterification (DEs) and towards pectin associated to intact plant cell wall [59].

### CBM motif involved in cardoon biomass deconstruction

Cellulases and hemicellulases catalytic domains are physically linked to CBM modules which participate in protein–carbohydrate interactions and facilitate the recognition and breakdown of PCWPs [60]. A significantly higher expression in CRD samples of the fungal CBM18 and *Ruminococcus* CBM37 families was observed in our study (Fig. 9). While the CBM37 is recognized as an important component of the *Ruminococcus* CAZy repertory because coordinates the cellulosome-independent fibrolytic activity of secreted carbohydrase [61], the fungal CBM18s are not directly involved in the recognition of PCWPs. Indeed, the CBM18 is a family of chitin-binding modules that can bind and protect the fungal cell wall from the catalytic action of chitinases [62]. Little is known about the physiological role of CBM18 modules in rumen fungi but a recent study suggests that these modules may play a role in fungal–methanogen physical associations, and fungal cell wall development and remodeling [63]. Therefore, in the context of PCWPs deconstruction, the CBM18 is not involved in the recognition and degradation of PCWPs but rather linked to the capacity of rumen fungi to colonize and compete against other microbes in the colonization of the cardoon biomass. The presence of an active growing mycelia in association with the cardoon biomass is further supported by the overexpression of *Neocallimastigaceae* Chitin-synthase coding transcripts in the CRD-enriched microbial community (Additional file 1: Table S13) [64].

### The role of carbohydrate esterases in the reduction of cardoon biomass recalcitrance

Depending on the kind of biomass, xyloglucans, xylans, mannans, glucomannans, and β-glucans are the major components of the hemicellulose, the second most abundant component of the plant cell wall [65–67]. We did not characterize the hemicellulose composition of cardoon biomass used in this work. However, a previous study indicates that cardoon hemicellulose is composed of xylans, arabinans, galactans, and, only in a small percentage, mannans [68]. The breakdown of hemicellulose requires the combined action of different fibrolytic enzymes, such as acetyl xylan esterases, feruloyl and ferulic acid esterases, endoxylanases, and arabinofuranosidases, and starts with the removal of acetyl residues of the xylan backbone [66]. Furthermore, the hydrolysis of ferulic acids ester bonds cross-linking hemicellulose to lignin also contributes to the reduction of biomass recalcitrance by exposing the PCWPs to the enzymatic action of fibrolytic enzymes [69, 70]. While CE1 is the only family including the feruloyl esterases [70], the majority of CE coding transcripts identified in our metatranscriptome were acetyl xylan esterases of the family CE6 which catalyzes the release of acetic acid from acetylated xylan (Additional file 1: Table S9) [70]. None of the CE6 transcripts resulted significantly more expressed in CRD samples (Fig. 9).

## Conclusions

The metatranscriptomic analysis of the cow rumen microbial communities enriched on cardoon lignocellulosic biomass, reported in this study, has provided a catalog of diversified carbohydrate-active enzymes potentially exploitable for the pretreatment of the lignocellulosic fraction of cardoon. Our data demonstrate that only a limited number of fibrolytic bacteria, namely, *Lachnospiraceae* and *Treponemataceae,* were able to persist up to 96 h in association with the most recalcitrant fraction of the cardoon fibres. However, further analysis will be necessary to test saccharification efficiency of such enzymes/microbes on cardoon PCWPs to validate their possible biotechnological applications. A different experimental and sequencing strategy will be also necessary to better explore the real contribution of rumen Eukaryotes to the degradation of cardoon PCWPs. While bacteria represent the most prominent fraction of the rumen microbiota, Eukaryotes, and in particular anaerobic fungi, are considered one of the best degraders plant polysaccharides. Our study did not specifically target the eukaryotic RNA via poly(A) enrichment, and as a consequence, we are likely underestimating the eukaryotic fraction of the rumen microbiota enriched in the cardoon nylon bags.

In conclusion, the cardoon fibre-adherent microbial community characterized in this study via metatranscriptomic analysis has the potential to be used in a range of biotechnological processes including biofuels, added value bioproducts and biomaterials for the biorefinery using cardoon biomass as renewable feedstock.

## Materials and methods

### Animal experiment

All animal procedures were approved by the Animal Care and Use Committee of the Università Cattolica del Sacro Cuore (Piacenza, Italy) and were strictly in accordance with the University’s guidelines for animal research.

Three ruminally cannulated dairy heifers (body weight = 763 ± 106 kg) were used in this study. Each animal was fed a diet based on 6 kg day^−1^ of grass hay, 2 kg day^−1^ of cardoon, and 1 kg day^−1^ of concentrate (concentrate composition: decorticated sunflower, 15.0%; soybean, 44%; wheat bran, 10.4%; CaCO_3_, 2.6%; MgO, 1.2%; NaCl, 0.6%; NaHCO_3_, 2.4; CaHPO_4_^.^2(H_2_O), 1.8%) for 6 weeks before starting the in vivo trial. See Additional file 1: Table S1 for a detailed composition of animal diet. A mix of microminerals and vitamins was supplemented to the diet (Additional file 1: Table S15). Animals were fed twice a day, at 07:00 and 19:00, with the same amount of feed to minimize the fluctuation of feed intake on the rumen microbial community composition. Animals had free access to water.

### Rumen and biomass-containing nylon bags sample collection

Cardoon plants growing under natural field conditions older than 3 years were harvested from the bioenergy farm of the Novamont S.p.A (Novara, Italy) in 2016. Cardoon biomass was supplied as part of the REBIOCHEM project consortium. The biomass was air-dried and ground to pass a 2 mm sieve using a Wiley mill. The ground material (10 g) was weighed into individual nylon bags with a mesh size of 50 µm and incubated in the rumen of three cannulated heifers. Digested cardoon biomass in nylon bags was collected through the rumen fistula after 48 and 96 h incubation time. In addition, digested fibers were collected from the rumen of each heifer before the early morning feeding. The samples were quickly washed in a sterile phosphate buffer solution, and the liquid excess was removed. The samples were immediately stored at -80 °C, until RNA extraction.

### Fiber degradation analysis: in situ digestibility and chemical composition of cardoon biomass

Air-dried test cardon previously milled through a 1-mm screen using a Wiley knife mill were weighed into 10 × 20 cm polyester bags (R1020 Forage Bag, ANKOM Technology, Macedon NY, US) with a pore size of 50 microns at a rate of 20 mg/cm2 of the nylon bag area as suggested by Diao er al., 2020 [71]. The bags were incubated in sextuple in the rumen for 48 and 96 h. Bags corresponding to each incubation time were recovered, and three of them rinsed four times in physiological solution and three times in phosphate buffered saline solution, gently hand squeezing them after each step. At the end of the in situ experiment, the rumen content was collected from three sites (cranial sac, dorsal sac, and ventral sac), pooled, and placed in the nylon bags. These nylon bags were then treated as for the cardoon residues, i.e., washed with physiological and PBS solution, and gently hand-squeezed after each washing step to remove the excess fluid. After the last washing step, the bags containing the solid fraction of the rumen and the cardoon were immediately frozen on dry ice and then kept at − 80 °C until total RNA extraction. The other six cardoon nylon bags representing 48 and 96 h incubation time (3 × 48 h and 3 × 96 h) were recovered, rinsed in a washing machine for 5 min, hand squeezed, oven dried at 60 °C to constant weight, and finally weighed to calculate the residual dry matter. Neutral detergent fiber (NDF), acid detergent fiber (ADF), and acid detergent lignin (ADL) were determined on the residues using the procedures described in [72]. The fiber composition analysis before and after 48 and 96 h incubation time is reported in Table 1.

### Total RNA extraction

Solid samples were pulverized in liquid nitrogen. Total RNA was extracted from 160 mg of cardoon biomass using the RNeasy PowerMicrobiome kit (MO BIO Laboratories, Carlsbad, CA, USA) according to the manufacturer’s protocol. Total nucleic acids were subjected to DNAse treatment following manufacturer instructions. In addition, the complete removal of DNA was verified by PCR with primers targeting the 16S rDNA gene. RNA integrity was assessed on an Agilent Bioanalyzer 2100 (Agilent Technologies, Palo Alto, CA, USA). RNA preparations used for subsequent next-generation sequencing (NGS) library preparation had an RNA integrity number (RIN) of ≥ 6.0. Aliquots of total RNA (2 µg each) were individually resuspended in RNAse-free water.

### 16S amplification and illumina MiSeq sequencing

One µg of total RNA was retrotranscribed using the cDNA high-capacity reverse transcription kit (Applied Biosystems, Foster City, CA, USA). The cDNA was diluted 1:40 in nucleic acid-free water and then used as a sample to produce 16S libraries according to the Illumina 16S metagenomic sequencing library protocol using PCR primers targeting a fragment of the 16S rRNA gene (V3–V4 region). Equimolar amounts of the generated libraries were sequenced on an Illumina MiSeq platform using MiSeq reagent kits following the 2 × 300-bp paired-end sequencing protocol. Approximately 400.000 reads per sample were produced.

Read analysis was conducted using DADA2 in the QIIME2 v2022.2 pipeline [73, 74]. The resulting amplicon sequence variants (ASVs) were classified against a pre-trained classifier (link: https://data.qiime2.org/2022.2/common/silva-138-99-nb-weighted-classifier.qza; last accessed on July 2022) based on the bacterial and archaeal SILVA database v138.1 [75]. The R package "phyloseq” [76] v1.38.0 was used in conjunction with microbiomeMarker v1.0.2 [77]. The alpha diversity indices Chao1 and Shannon were calculated using phyloseq, while microbiomeMarker was used to perform an ANOVA-like differential abundance analysis [78–80] of ASVs applying a *p* values correction for multiple testing using the Benjamini–Hochberg (BH) false discovery rate (FDR) procedure (FDR < 0.01).

### Illumina HiSeq RNA sequencing

The amount of total RNA used for sequencing was 1 µg. To maximize mRNA representation in the metatranscriptomic libraries, the Ribo-Zero rRNA removal kit (Bacteria) (Illumina, San Diego, CA, USA) was used to remove rRNAs from the total RNA preparations. Library preparation was performed using the Illumina TruSeq stranded total RNA kit (Illumina). Sequencing was carried out using an Illumina HiSeq2000 platform. In total, 178 million of paired-end reads were generated from nine independent libraries: three libraries from rumen solid samples, three libraries from cardoon biomass digested for 48 h, and three libraries from cardoon biomass digested for 96 h.

### De novo metatranscriptome assembly and annotation

A de novo co-assembly was used to assemble RNA-Seq reads from CRD and RMN samples into transcripts using Trinity v2.14.0 [81]. Only transcripts having a minimum length of 500 bp were used for downstream analyses. Mmseq2 v13.45111 2bLCA (low common ancestor) protocol [82] was used for the taxonomic classification of transcripts with the option “–orf-filter 0”, to disable the rejection of short-query transcripts when performing searches against the NCBI non-redundant (NR) protein database (https://ftp.ncbi.nlm.nih.gov/blast/db/FASTA/; last accessed in August 2022) The module “mmseqs search” was used for the annotation of transcripts against the carbohydrate-active enzymes (CAZy) database v8 [83] (http://bcb.unl.edu/dbCAN2/download/; last accessed in August 2022) with the following option: “-s 7.5 –alignment-mode 3 –min-seq-id 0.35 –cov 0.6”. Finally, eggNOG-mapper v2.1.9 and the eggNOG database v5.0.2 [84] (http://eggnog5.embl.de/download/emapperdb-5.0.2/; last accessed in August 2022) were used to annotate transcripts against phylogenetically refined orthologous groups (seed orthologues), and against the functional KEGG orthologs (KO) with the following options “–itype metagenome –genepred search –sensmode ultra-sensitive –query_cover 60 –pident 40”.

### Metatranscriptomic data analysis

All metatranscriptomic data were analyzed using the R statistical software v4.1.3. Transcript quantification was performed with Salmon v1.3.0 [85]. Salmon quant files were imported into R using tximport v1.22.0 [86], rRNA transcripts were removed using SortMeRNA v4.3.6 [87], and transcripts abundance data were adjusted for the effect of transcript length. The “adonis2” function from the package vegan v2.6–2 [88] was used to perform a permutational univariate analysis of variance on Bray–Curtis dissimilarities calculated using the Phyloseq “distance” function [76]. For differential abundance analysis between CRD and RMN samples, the transcripts abundance data adjusted for the effect of transcript length were binned into seed orthologs based on eggNOG annotation. The seed orthologs count table was finally analyzed with edgeR v3.36.0 [89] based on quantile-adjusted conditional maximum likelihood method. Differential abundance of seed orthologs was considered significant for FDR-adjusted *p* values < 0.01. The same statistical approach was used to perform a within-taxon differential expression analysis for transcripts affiliated to *Prevotellaceae*, *Fibrobacteraceae*, *Lachnospiraceae*, *Neocallimastigaceae*, *Oscillospiraceae*, and *Treponemataceae*. Transcripts were considered differentially expressed between CRD and RMN samples for FDR-adjusted *p* values < 0.01.

### Metabolic potential of CRD and RMN microbial community and functional enrichment

KO and KEGG modules associated with transcripts via eggNOG annotation were used to identify transcripts involved in methanogenesis, degradation of pectin, and synthesis of SCFAs acetate, butyrate, and propionate. Butyrate production was determined by searching for transcripts annotated as phosphate butyryltransferase (K00634; *ptb*), butyrate kinase (K00929; *buk*), acetate CoA/acetoacetate CoA-transferase alpha subunit (K01034; *atoD*), acetate CoA/acetoacetate CoA-transferase beta subunit (K01035; *atoA*), and acetate CoA-transferase (K19709; *ydiF*). KOs used for the identification of transcripts involved in the synthesis of propionate were: methylmalonyl-CoA mutase (K01847; MUT), methylmalonyl-CoA mutase, N-terminal domain (K01848; *mcmA1*), methylmalonyl-CoA mutase, C-terminal domain (K01849; *mcmA2*), methylmalonyl-CoA decarboxylase (K11264; *mmcD*), and methylmalonyl-CoA/ethylmalonyl-CoA epimerase (K05606; MCEE). Transcripts annotated as acetate-CoA ligase (ADP-forming) subunit alpha (K01905; *acdA*), acetate-CoA ligase (ADP-forming) subunit beta (K22224; *acdB*), and acetate-CoA ligase (ADP-forming) (K24012; *acdAB*) were used for assessment of acetate production. Finally, the capacity to break down pectin was evaluated by searching for transcripts annotated in the pectin degradation KEGG module M00081. To identify the major methanogenic pathways occurring in our metatranscriptomic data, we searched for transcripts involved in methane synthesis from acetate (K00193; K00194; K00197), methanol (K04480; K14080; K14081), methylamines (K14083; K14082; K14084; K16178; K16179; K16177; K16176), and CO_2_ (K00200; K00201; K00202; K00203; K11261; K00205; K11260; K00204; K00672; K01499; K00319; K13942; K00320). The R package clusterProfiler v4.2.2 [90] was used to identify over-represented KEGG modules in clusters of differentially abundant seed orthologs. KEGG modules were considered significantly enriched for FDR-adjusted *p* values < 0.05.

### Supplementary Information

**Additional file 1: Table S1.** Composition of the ingredients and calculated composition of the diets fed to cannulated heifers during the trial;** Table S2. **Chemical composition of the cardoon residue in the nylon bags, rumen solid fraction, and undigested cardoon biomass;** Table S3. **Relative abundance and prevalence of amplicon sequence variants (ASVs) in cardoon (CRD) and rumen (RMN) samples; **Table S4.** Alpha diversity estimators for cardoon (CRD) and rumen (RMN) samples;** Table S5.** Differentially abundant amplicon sequence variants (ASVs) predicted via GLM–ANOVA and k-mean clustering results;** Table S6.** MMseqs2 LCA taxonomy report used to generate the Sankey plot in Pavian; **Table S7.** eggNOG annotation of transcript in RMN- and CRD-adherent microbial community; **Table S8.** MMseqs2 transcriptome annotation against the Carbohydrate active enzymes (CAZymes) database;** Table S9.** CAZy families frequencies;** Table S10.** Transcripts associated to differentially expressed seed orthologs; **Table S11.** KEGG module enrichment analysis of transcripts associated to differentially expressed seed orthologs; **Table S12.** Differentially expressed transcripts within major fiber degrading Taxon; **Table S13.** Differentially expressed CAZy transcripts within major fiber degrading Taxon; **Table S14.** Over-representation analysis (ORA) results of the CAZy families over-represented within the CAZy-coding transcripts more expressed in the CRD-enriched microbial community; **Table S15.** Micromineral and vitamin content supplemented to the fed.

**Additional file 2: **Taxonomic composition and relative abundances of transcripts involved in the methylotrophic and hydrogenotrophic methanogenic pathways. Relative abundances were calculated from the total count of reads mapping to the metatranscriptome-assembled transcripts involved in methanol (A), H_2_/CO_2_ (B), and methylamine (C) methanogenic pathways.

## Data Availability

The data sets supporting the conclusions of this article are available in the NCBI Short Read Archive repository under the BioProject accession number PRJNA659627. The metatranscriptome assembled transcripts and the R codes used in our analyses are available in the Figshare repository, doi: 10..6084/m9.figshare.22680448.

## Abbreviations

ADF: Acid detergent fiber
ADL: Acid detergent lignin
ASV: Amplicon sequence variants
BLAST: Basic local alignment search tool
CAZy: Carbohydrate-active enzymes
CBM: Carbohydrate binding modules
CE: Carbohydrate esterase
CPM: Count per million
CRD: Cardoon
DR: Dry matter
FDR: False discovery rate
GH: Glycoside hydrolase
GT: Glycosyl transferase
KEGG: Kyoto encyclopedia of genes and genomes
KO: KEGG orthology
LCA: Low common ancestor
LFC: Log_2_ fold change
NGS: Next-generation sequencing
NCBI: National center for biotechnology information
NDF: Neutral detergent fiber
NR: Non-redundant
OTU: Operational taxonomic unit
PBS: Phosphate buffer solution
PC: Principal component
PCWPs: Plant cell wall polysaccharides
PL: Polysaccharide lyases
qCML: Quantile-adjusted conditional maximum likelihood
RMN: Rumen
SdO: Seed orthologs
