# Supplementary figures and images for: Structural and functional analysis of the active cow rumen’s microbial community provides a catalogue of genes and microbes participating in the deconstruction of cardoon biomass
Source: Biotechnol Biofuels Bioprod. 2024 Apr 8;17:53. doi: 10.1186/s13068-024-02495-4 (PMC11003169; doi:10.1186/s13068-024-02495-4)

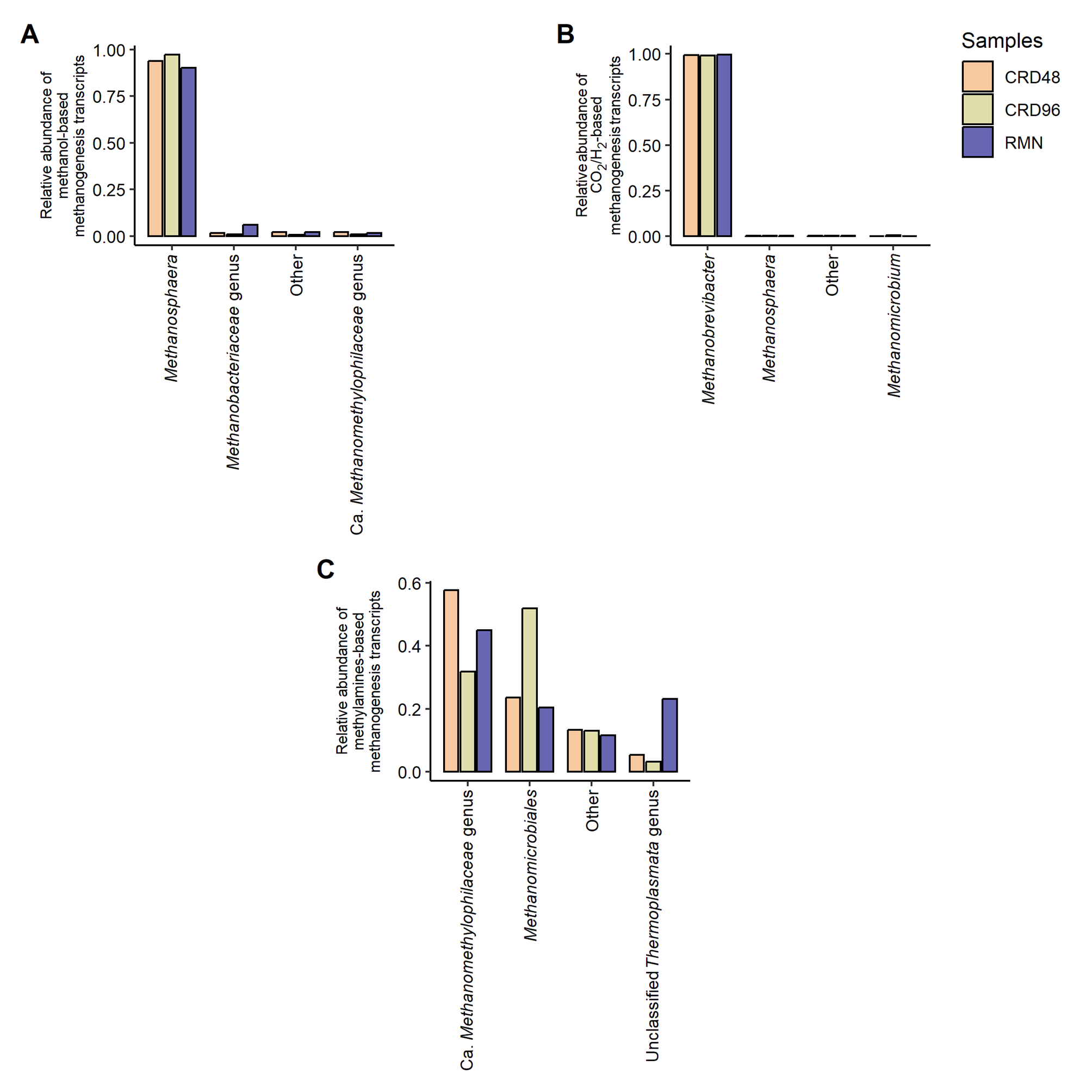

Supplement: Supplementary file 2 — Additional file 2: Taxonomic composition and relative abundances of transcripts involved in the methylotrophic and hydrogenotrophic methanogenic pathways. Relative abundances were calculated from the total count of reads mapping to the metatranscriptome-assembled transcripts involved in methanol (A), H2/CO2 (B), and methylamine (C) methanogenic pathways. [file 13068_2024_2495_MOESM2_ESM.tif]
